# Brief communication: body composition and hidden obesity in people living with HIV on antiretroviral therapy

**DOI:** 10.1186/s12981-024-00599-3

**Published:** 2024-03-01

**Authors:** K. Konishi, H. Nakagawa, T. Asaoka, Y. Kasamatsu, T. Goto, M. Shirano

**Affiliations:** 1 Department of Infectious Diseases, Osaka City General Hospital, Osaka, Japan; 2 Department of Oral Microbial Control, Graduate School of Medicine, Osaka University, 2-2 Yamadaoka, Suita-shi, Osaka, 565-0871 Japan; 3 Department of Infection Control, Osaka University Hospital, Osaka, Japan

**Keywords:** HIV, Antiretroviral therapy, Weight gain, Body composition, Japan

## Abstract

**Background:**

Increased incidence of lifestyle diseases as side-effects of antiretroviral therapy (ART) have been reported in people living with HIV (PLWH). Few studies have evaluated obesity and hidden obesity in Japanese PLWH and their association with ART. In order to provide more appropriate drug selection and lifestyle guidance, we investigated the relationship between the effects of HIV infection and ART on the body composition of Japanese PLWH.

**Methods:**

PLWH who visited the outpatient clinic and had body composition measured using the body composition analyzer InBody 570 were included in this study. Medications, comorbidities, and blood test data were obtained. Body mass index (BMI), body fat percentage, and skeletal muscle mass index (SMI) were measured.

**Results:**

In this study, 543 patients were included. Based on body shape, patients were classified into a thin group (13), normal weight group (14), hidden obesity group (158), apparent obesity group (14), and obesity group (218). Compared with the normal weight group, the hidden obesity group had a higher prevalence of comorbidities and a lower SMI.

**Conclusions:**

PLWH are more likely to have obesity than the general population, indicating that hidden obesity is common even among those with a normal BMI. It is important to measure body fat percentage along with body weight, as hidden obesity can be missed. Further investigation of the effects of ART on body composition is needed.

## Background

Although antiretroviral therapy (ART) extends the lives of people living with human immunodeficiency virus (HIV) (PLWH), its potential to induce weight gain and metabolic disorders necessitates further exploration, particularly regarding hidden obesity. In this condition, body fat percentage is high despite a normal body mass index (BMI) [1]. Integrase strand transfer inhibitors (INSTI), such as dolutegravir (DTG), and nucleoside analog reverse transcriptase inhibitors, such as tenofovir alafenamide (TAF) and emtricitabine (FTC), have been recommended as the key drugs and backbone of first-line treatment owing to their efficacy and minimal serious side effects [2, 3]. However, weight gain associated with these drugs highlights the need for further research, especially regarding body composition changes in Asian PLWH, a less studied group.

Body composition is classified into body fat, bone, and lean soft tissue (mainly muscle and water), and imbalances among these components are associated with various lifestyle diseases and chronic disease symptoms [4–6]. Subsequently, anthropometric measurements, including body water content, lean body mass, muscle mass, and body fat mass, are obtained. Reports on weight gain in PLWH have mainly investigated changes in body weight and BMI, with few reports investigating body composition. Hidden obesity, defined by high body fat with normal BMI, raises concerns due to visceral fat accumulation around abdominal organs. Recent studies reveal a link between hidden obesity and increased cardio-metabolic risks, including metabolic syndrome, insulin resistance, and factors elevating cardiovascular disease risk [7]. Thus, assessing hidden obesity via anthropometric methods to efficiently address these health concerns is imperative. In this study, we used InBody to investigate the status of obesity and hidden obesity in Japanese PLWH and the effects of HIV infection and ART on body composition to provide more appropriate drug selection and lifestyle guidance.

## Methods

### Study design and population

We conducted an observational study at a core acquired immunodeficiency syndrome (AIDS) base hospital in Japan, including male PLWH who visited the outpatient clinic between July 1, 2021 and June 30, 2022. We included patients whose body composition could be measured using the InBody 570 (InBody Japan Co. Ltd., Tokyo, Japan). The exclusion criteria were less than 12 weeks post-ART change at the time of InBody 570 measurement and multiple key drug prescriptions. The study was approved by the corresponding institutional review board committee as a single-center study (Approval Number 2205020) and conducted in compliance with the Declaration of Helsinki.

### Measurements

The body composition was measured using the InBody 570 body composition analyzer. Height, weight, BMI, body fat percentage, skeletal muscle index (SMI), and visceral fat level (truncated one-digit value of visceral fat cross-sectional area estimated from impedance) were measured. BMI was classified based on the obesity criteria of the Japan Society for the Study of Obesity [7]: underweight (< 18.5 kg/m^2^), normal (18.5–25 kg/m^2^), and obese (> 25 kg/m^2^). Body fat percentages of 10–20% and ≥ 20% were defined as normal and high, respectively. The body shape of the patients was classified based on their BMI and body fat percentage data into thin group, normal weight group (NWG), hidden obesity group (HOG), apparent obesity group, and overweight obesity group (OOG). Patient clinical data included age, AIDS history, nadir CD4 count, HIV diagnosis duration, ART duration, and current medication use for hypertension, diabetes, and dyslipidemia. Blood analysis covered CD4-positive T lymphocyte count, HIV-RNA level, aspartate aminotransferase (AST), alanine aminotransferase (ALT), gamma-glutamyl transferase (γGTP), high-density lipoprotein cholesterol (HDL-C), low-density lipoprotein cholesterol (LDL-C), triglyceride (TG), HbA1c level, and C-reactive protein (CRP) level.

### Statistical analysis

Body shape was classified into five categories based on BMI and body fat percentage, and their distribution by age group was examined. Body composition was compared among the three groups (NWG, HOG, and OOG). For continuous variables, the Kruskal–Wallis test was used to compare differences between the three groups. For categorical variables, including participant characteristics, the Chi-square test or Fisher’s exact test was used for comparisons. The Bonferroni method was used as a multiple comparison method for body composition items that showed significant differences among the three groups. Statistical significance was set at a two-sided P < 0.05. All statistical analyses were performed using IBM SPSS, version 28.0 (IBM, Armonk, NY, USA). Clinical data expressed as continuous variables were described as median and interquartile range [first quartile–third quartile].

## Results

The body composition of 568 patients was measured. After excluding 11 patients who had been on ART for less than 12 weeks and 13 patients who were taking multiple key drugs with different mechanisms of action, 543 patients were included. The median age was 48 years [40.5–55.5]. As per BMI, 13 (2.4%), 298 (54.9%), and 232 (54.9%) were considered underweight, normal, and obese, respectively. The most common ART used was bictegravir/TAF/FTC (n = 131), followed by DTG + TAF/FTC (n = 97) and raltegravir + TAF/FTC (n = 80). TAF/FTC.

### Body composition measurement

Distribution of body size groups classified by age.

**Fig. 1:**
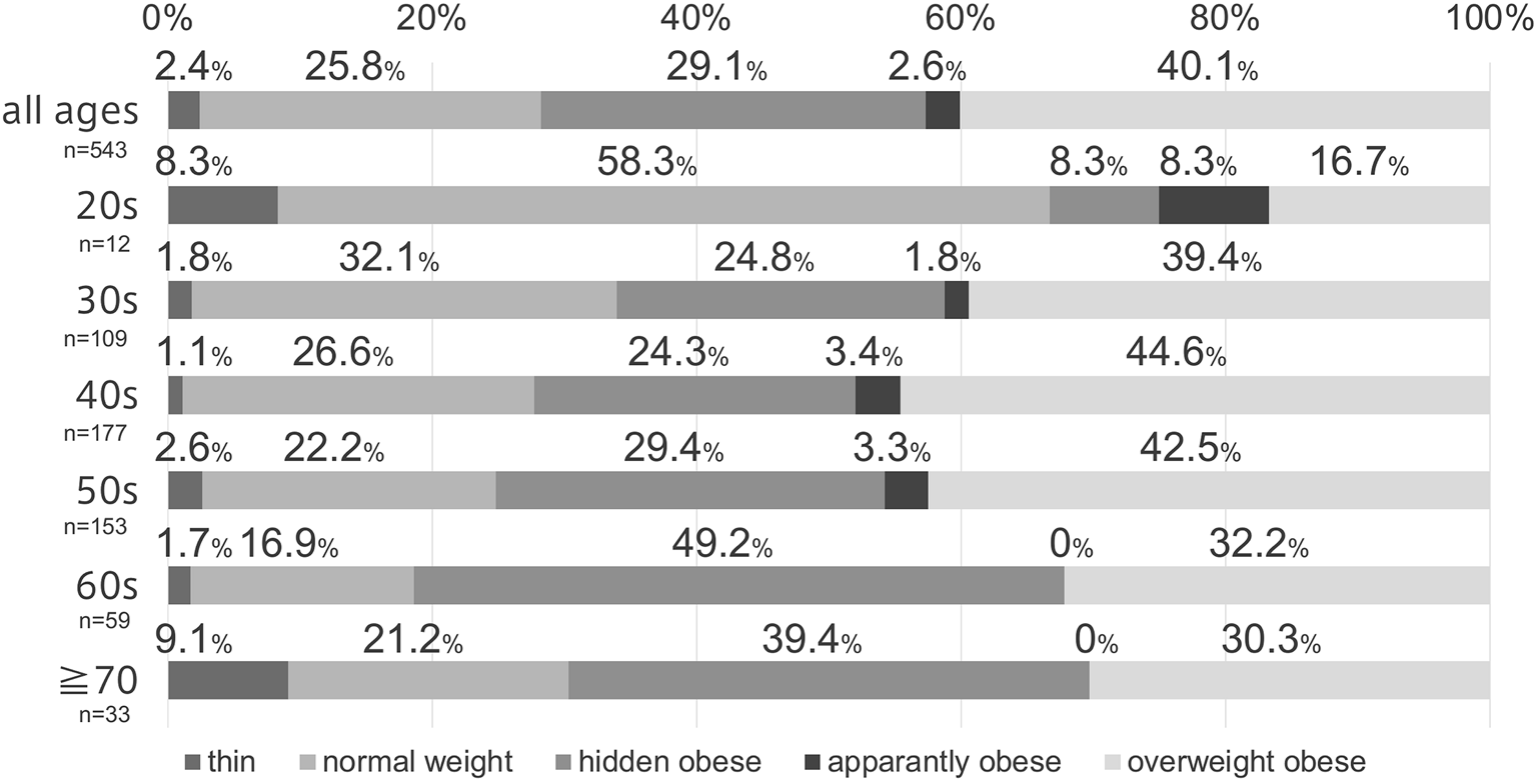
Distribution of the body shape of the patients classified by age group Thin group: body mass index (BMI) < 18.5 kg/m^2^; normal group: BMI 18.5–25 kg/m^2^ and body fat percentage < 20%; hidden obesity group: BMI ≥ 18.5 kg/m^2^ or < 25 kg/m^2^ and body fat percentage ≥ 20%; apparently obesity group: BMI ≥ 25 kg/m^2^ and body fat percentage < 20%; obesity group: BMI ≥ 25 kg/m^2^ and body fat percentage ≥ 20% BMI, body mass index

Figure 1 shows the distribution of the patients according to their body size classified by age group. The percentage of NWG tended to decrease with age. In contrast, the percentage of HOG tended to increase with age. Meanwhile, OOG showed a decreasing trend after reaching a peak in the 40 s.

### Comparison of background and body composition by body shape

Table 1 shows the comparison of the patient background and body composition of 143, 158, and 218 patients in the NWG, HOG, and OOG, respectively. Significant differences were noted in age, hypertension, and lipid disorder treatment in patient backgrounds. Regarding body composition, significant differences were observed in SMI and visceral fat levels. In the multiple comparison method, SMI and visceral fat levels showed significant differences between the NWG and HOG, the HOG and OOG, and the NWG and OOG. Visceral fat levels increased significantly from the NWG to the HOG and OOG; however, SMI was lower for the OOG, NWG, and HOG in that order. The blood and biochemical laboratory results showed significant differences in AST, ALT, γ-GTP, HDL-C, TG, HbA1c, and CRP levels. AST and ALT levels were significantly higher in OOG than in NWG and HOG, with γ-GTP showing a rising trend from NWG to OOG. HDL-C levels decreased, and TG levels increased in the same direction, with HbA1c being higher in the OOG group than in the other groups. Similarly, CRP levels followed an upward trend from NWG to OOG.

**Table 1:** Characteristics and anthropometric values stratified by BMI and body fat percentage

	NWG (n = 140)	HOG (n = 158)	OOG (n = 218)	*P*
Age (years)	47 [38–52]	51 [42–60]	48 [41–54]	< 0.001
History of AIDS diagnosis	49 (35.0%)	51 (32.3%)	86 (39.4%)	0.344
Nadir CD4 (/µL)	145 [60–291]	149 [50–240]	149 [42–251]	0.528
Duration since HIV diagnosis (years)	8 [4–14]	10 [5–13]	9 [5–13]	0.500
Duration of ART (months)	93 [45–141]	104 [57–148]	95 [58–130]	0.597
Use of TAF	101 (72.1%)	119 (75.3%)	165 (75.7%)	0.732
Key drug				0.188
INSTI	120 (85.7%)	130 (82.3%)	176 (80.7%)	
PI	16 (11.4%)	17 (10.8%)	21 (9.6%)	
NNRTI	4 (2.9%)	11 (7%)	21 (9.6%)	
Hypertension treatment	16 (11.4%)	24 (15.2%)	50 (22.9%)	0.013
Diabetes treatment	5 (3.6%)	12 (7.6%)	23 (10.6%)	0.055
Lipid disorder treatment	2 (1.4%)	18 (11.4%)	38 (17.4%)	< 0.001
SMI(kg/m^2^)	7.7 [7.2–8.1]	7.4 [7.1–7.7]	8.4 [8.0–8.9]	< 0.001
Visceral fat level	4 [3–5]	6 [6, 7]	10 [8–13]	< 0.001
CD4^+^ cell count (/µL)	577 [414–725]	577 [448–771]	608 [482–819]	0.056
HIV-RNA level (copies/mL)	20 [20–20]	20 [20–20]	20 [20–20]	0.679
AST (U/L)	22 [19–25]	22 [18–27]	25 [21–34]	< 0.001
ALT (U/L)	19 [15–24]	19 [15–27]	30 [21–49]	< 0.001
γGTP (U/L)	20 [15–31]	29 [19–54]	39 [24–67]	< 0.001
Creatinine (mg/dL)	0.92 [0.83–1.00]	0.88 [0.78–1.00]	0.91 [0.82–1.03]	0.081
HDL-C (mg/dL)	59 [48–71]	51 [44–62]	49 [42–57]	< 0.001
LDL-C (mg/dL)	109 [92–136]	116 [97–139]	121 [103–142]	0.086
TG (mg/dL)	117 [81–157]	147 [95–208]	182 [122–262]	< 0.001
HbA1c (%)	5.5 [5.3–5.6]	5.5 [5.3–5.8]	5.7 [5.4–6.1]	< 0.001
CRP (mg/dL)	0.03 [0.03–0.07]	0.04 [0.03–0.11]	0.08 [0.04–0.19]	< 0.001

Data are presented as counts (percentages) or median [interquartile range]

*BMI* body mass index, *AIDS* acquired immunodeficiency syndrome, *HIV* human immunodeficiency virus, *ART* antiretriviral therapy, *NWG* normal weight group (BMI 18.5–25 kg/m^2^ and body fat percentage < 20%); *HOG* hidden obesity group (BMI ≥ 18.5 kg/m^2^ or < 25 kg/m^2^ and body fat percentage ≥ 20%), *OOG* overweight obesity group (BMI ≥ 25 kg/m^2^ and body fat percentage ≥ 20%), *TAF* tenofovir alafenamide, *INSTI* integrase strand transfer inhibitor, *NNRTI* non-nucleoside reverse transcriptase inhibitor, *PI* protease inhibitor, *BMI* body mass index, *SMI* skeletal muscle mass index, *AST* aspartate aminotransferase, *ALT* alanine aminotransferase, *γGTP* gamma-glutamyl transferase, *HDL-C* high-density lipoprotein cholesterol, *LDL-C* low-density lipoprotein cholesterol, *TG* triglyceride, *CRP* C-reactive protein

P*: Normal group vs. hidden obesity group vs. obesity group; Kruskal–Wallis test for continuous variables, χ^2^ test or Fisher’s exact test for other variables. The Bonferroni method was used as a multiple comparison method for the body composition items that showed significant differences among the three groups

## Discussion

This study serves as an initial comprehensive analysis of body composition in Japanese men with HIV over the age of 20 at a single center. Notably, our findings reveal a prominent rate of hidden obesity, accounting for a considerable proportion of the standard obesity rate (40.1%), underscoring the complex impact of ART on body composition. In Japan, obesity is defined as having a BMI ≥ 25 kg/m^2^, following a study [8] that highlighted the increased prevalence of hypertension, lipid disorders, hyperglycemia, and other conditions [9]. According to the National Health and Nutrition Survey conducted in Japan in 2019 [10], the prevalence of obesity among males was 23.1%, 29.4%, 39.7%, 39.2%, 35.4%, and 28.5% for those in their 20 s, 30 s, 40 s, 50 s, 60 s, and > 70 years, respectively.

This study found that 29.1% of participants and 53.0% in the normal BMI group had hidden obesity. Additionally, hypertension, diabetes, and lipid disorders were more prevalent among those with hidden or apparent obesity than in normal-weight individuals, indicating the importance of lifestyle guidance regardless of BMI.

The study further highlights the unique challenge of hidden obesity surpassing overt obesity in individuals with PLWH aged older than 60 years, signaling the risk of a surge in obesity rates within this group in the future. Although there is no clear mechanism for ART-induced weight gain, inflammatory markers, such as IL-6 and CRP, are more elevated in PLWH than in non-PLWH, even when the virus is well-controlled with ART [11]. These inflammatory markers cause insulin resistance and are associated with lifestyle diseases such as weight gain and diabetes [5]. In this study, CRP levels increased significantly from the NWG to the HOG to the OOG, suggesting an association between weight gain and chronic inflammation.

Classifying the body shapes (hidden obesity, normal, obesity) of Japanese individuals based on BMI and body fat percentage revealed that pulse wave velocity (an arterial stiffness indicator), HDL-C, LDL-C, and adiponectin significantly increased in the hidden obesity and obesity groups compared to the normal group, with no significant differences between the hidden and obesity groups [12]. These findings suggest that the prevention of lifestyle diseases is as important for patients with hidden obesity as it is for patients with obesity; however, it may be overlooked if body fat percentage is not measured.

The cross-sectional design limits linking body composition changes directly to ART. Future research should use longitudinal designs, include dietary analysis, and account for ART history to explore ART's comprehensive effects on body composition. These findings highlight the need for further studies with nutritional evaluations and interventions to improve PLWH's long-term health. This summary underscores the importance of advanced nutritional and lifestyle interventions in PLWH management, situating the study's implications in wider public health and clinical contexts.

## Data Availability

The data that support the findings of this study are available from the corresponding author upon reasonable request.

## Abbreviations

ART: Antiretroviral therapy
HIV: Human immunodeficiency virus
PLWH: People living with HIV
NNRTI: Non-nucleoside analogue reverse transcriptase inhibitors
PI: Protease inhibitors
INSTI: Integrase strand transfer inhibitors
DTG: Dolutegravir
TAF: Tenofovir alafenamide
FTC: Emtricitabine
BMI: Body mass index
AIDS: Acquired immunodeficiency syndrome
SMI: Skeletal muscle index
NWG: Normal weight group
HOG: Hidden obesity group
OOG: Overweight obesity group
AST: Aspartate aminotransferase
ALT: Alanine aminotransferase
γGTP: Gamma-glutamyl transferase
HDL-C: High-density lipoprotein cholesterol
LDL-C: Low-density lipoprotein cholesterol
TG: Triglyceride
CRP: C-reactive protein
